# Clinical characterization of an *emm1*-dominant group A *Streptococcus* outbreak in Chile in the context of the previous 6 years

**DOI:** 10.3389/fcimb.2026.1682478

**Published:** 2026-02-25

**Authors:** Gonzalo Valenzuela, Patricia García, Juan A. Ugalde, Nicolás Canales, Javiera Jiménez, Tomás Carrasco, Patricio Ross, Pamela Medina, Carolina Núñez, Aniela Wozniak

**Affiliations:** 1 Department of Pediatric Infectious Diseases and Immunology, Escuela de Medicina, Pontificia Universidad Católica de Chile, Santiago, Chile; 2 Red de Salud UC CHRISTUS, Santiago, Chile; 3 Department of Clinical Laboratories; Laboratory of Microbiology, Escuela de Medicina, Pontificia Universidad Católica de Chile, Santiago, Chile; 4 Millennium Initiative for Collaborative Research on Bacterial Resistance (MICROB-R), Santiago, Chile; 5 Center for Bioinformatics and Integrative Biology, Facultad de Ciencias de la Vida, Universidad Andrés Bello, Santiago, Chile; 6 Escuela de Medicina, Pontificia Universidad Católica de Chile, Santiago, Chile; 7 Department of Infectious Diseases, Escuela de Medicina, Pontificia Universidad Católica de Chile, Santiago, Chile

**Keywords:** group A *Streptococcus*, invasive infections, M1_UK_ lineage, outbreak, virulence factors, emm typing and genomic surveillance

## Abstract

**Introduction:**

In 2024, Chile experienced an outbreak of invasive group A *Streptococcus* (iGAS) infections. Although increasing reports of GAS outbreaks have been described worldwide, it remains unclear whether these events are driven by increased virulence of circulating lineages or by lineage replacement. In parallel, the burden of hospitalized noninvasive GAS infections (h-niGAS), which may present with severe disease, remains poorly characterized. This study aimed to compare epidemiological, clinical, antimicrobial resistance, and genomic features of iGAS and h-niGAS infections in Chile over a six-year period.

**Methods:**

We analyzed 2,307 GAS isolates collected between 2018 and 2024, including 2,249 noninvasive isolates and 58 iGAS isolates. Noninvasive cases were classified as outpatient niGAS or hospitalized niGAS (h-niGAS). Antimicrobial susceptibility testing was performed for all isolates. *emm* typing was performed on isolates from hospitalized patients (28 iGAS and 94 h-niGAS). Whole-genome sequencing was performed on outbreak-related isolates, followed by virulence gene profiling and phylogenetic analysis. Clinical characteristics were compared between iGAS and non-iGAS cases and across time periods.

**Results:**

Among niGAS isolates, 2,119 were obtained from outpatients and 130 from hospitalized patients. h-niGAS isolates showed the highest proportion of clindamycin resistance (42%), whereas iGAS isolates showed the lowest (16%). Notably, clindamycin resistance among iGAS increased from 0% during 2020–2023 to nearly 20% in 2024. The most frequent *emm* types were *emm1*, *emm12*, and *emm4*. The frequency of *emm1* increased from 0% in 2020–2023 to 10% in 2024, while overall *emm* diversity declined markedly (Simpson’s reciprocal index from 17.3 to 6.63). Clinically, iGAS infections were associated with immunosuppression, chronic liver disease, viral coinfection, and trauma. During the 2024 outbreak, iGAS cases did not differ clinically from those reported in 2018–2023; however, h-niGAS cases required more surgical procedures (p = 0.005) and medical evaluations (p = 0.025). Whole-genome sequencing revealed predominance of *emm1*, including three M1UK strains harboring all 27 defining single-nucleotide polymorphisms. *emm1* isolates carried a higher number of virulence-associated genes compared with non-*emm1* isolates (p < 0.05). Phylogenetic analysis showed close relatedness to strains from the United Kingdom, Argentina, and the United States.

**Discussion:**

These findings suggest that the Chilean outbreak was driven by the expansion of the *emm1* lineage rather than an increase in strain virulence. The identification of M1UK strains and the reduced *emm* diversity support a model of lineage introduction and clonal expansion. The significant clinical burden observed among h-niGAS cases underscores the importance of including hospitalized noninvasive infections in surveillance efforts. Ongoing integrated clinical and genomic surveillance of both iGAS and h-niGAS is essential to monitor emerging lineages and antimicrobial resistance trends.

## Introduction

Infections caused by *Streptococcus pyogenes*, also referred to as group A *Streptococcus* (GAS), can cause a broad spectrum of diseases, ranging from non-invasive infections (niGAS) to severe invasive infections (iGAS), such as streptococcal toxic shock syndrome or necrotizing fasciitis (Brouwer et al., 2023). The definition of iGAS is based on isolation from sterile sites, whereas niGAS are obtained from non-sterile sites (Jespersen et al., 2020). Although niGAS are generally considered less severe, certain cases require hospitalization and even surgical intervention (e.g., peritonsillar abscesses); they will be referred here as h-niGAS (“h” for hospitalized). Both iGAS and h-niGAS can be associated with substantial morbidity, prolonged treatment, and extended hospital stays.

Since 2022, several countries in Europe and North America have reported a significant rise iniGAS, mainly linked to the expansion of the hypervirulent *emm1* lineage, including the M1_UK_ clone (World Health Organization (WHO), 2022; Alcolea-Medina et al., 2023; van Kempen et al., 2023). This variant has 27 distinctive single-nucleotide polymorphisms (SNPs) and shows derepression of the superantigen toxin SpeA. Recent outbreaks have shown the spread of M1_UK_, along with other clones like M1_DK_ in Denmark, in a setting of decreased population immunity after COVID-19-related isolation measures were relaxed (Lynskey et al., 2019; Johannesen et al., 2023; Vieira et al., 2024). Genomic data on GAS clinical isolates from Latin America remain extremely limited, being currently restricted to reports from Argentina and Uruguay (Pan American Health Organization,; Cipolla et al., 2025), together with a single documented outbreak in Brazil (Fernandes et al., 2017). This scarcity of regional genomic surveillance data hampers the understanding of lineage circulation, virulence shifts, and outbreak dynamics in South America. In Chile, an increase in invasive infections was reported during the first 6 months of 2024 (Vigilancia de laboratorio de enfermedad invasora por Streptococcus pyogenes en Chile 2014-2023/junio 2024, 2024). It remains unclear whether this surge was attributable to heightened virulence of previously circulating lineages or if these lineages were replaced with different, more virulent ones. Although invasive GAS infections have been prioritized due to their severity, h-niGAS infections remain comparatively understudied despite their substantial clinical burden, frequent need for hospitalization or surgical intervention, and their relevance for antimicrobial resistance surveillance (Lapthorne et al., 2024). This comparative approach strengthens regional surveillance efforts and provides a more comprehensive view of GAS disease burden across clinical spectrums, highlighting the importance of integrated genomic and antimicrobial susceptibility surveillance to detect the expansion of hypervirulent lineages, monitor emerging clindamycin resistance, and inform outbreak preparedness.

We thoroughly characterized iGAS and h-niGAS isolates associated with the 2024 outbreak and conducted a comparative analysis with isolates collected from 2018 to 2023, focusing on *emm* type distribution, clindamycin resistance, and the clinical features of the infected patients. Our objective was to ascertain whether iGAS and h-niGAS occurring during the 2024 outbreak exhibited differences from those identified in the preceding years. Furthermore, we performed a genomic characterization of the iGAS and h-niGAS isolates obtained during the 2024 outbreak and compared them with other GAS isolates from Latin America and worldwide.

## Materials and methods

### Bacterial isolates and growth conditions

Experimental procedures were performed in accordance with the Ethics and Biosafety Committee of the Pontificia Universidad Católica de Chile (protocol ID 250130001). Clinical GAS isolates consecutively obtained from symptomatic patients (adult and children) between January 2018 and December 2024 at the Clinical Health Network UC-CHRISTUS (Santiago, Chile) were included in the study. The isolates were from outpatients (49 sampling units distributed nationwide) and inpatients (two tertiary hospital centers in the capital region receiving patients from all regions of the country). Although microbiological processing was centralized within the UC-CHRISTUS network in Santiago, the study population was not geographically restricted, as outpatient isolates originated from a nationwide network and inpatient cases corresponded to referrals from all regions of the country.

Specimens were seeded in sheep blood agar (bioMerieux, France), and beta-hemolytic colonies were identified using MALDI-TOF (Bruker, Germany). Only one isolate per patient was included. The isolates were stored in brain–heart infusion broth (Merck, Germany) supplemented with 5% sheep blood at -80°C and subcultured on Columbia blood agar (bioMerieux, France). Only isolates that successfully grew after thawing were included for downstream analyses, including *emm* typing or whole-genome sequencing. niGAS isolates were recovered from non-sterile sites: throat swabs, sputum, ear aspirates, peritonsillar abscesses, wounds, urine, endotracheal aspirates, and endocervical secretions. iGAS isolates were recovered from blood, pleural fluid, and biopsy tissues. Six GAS isolates obtained from non-sterile sites, namely, five wounds and one endotracheal aspirate, were clinically diagnosed as necrotizing fasciitis and pneumonia, respectively. Therefore, they were considered iGAS.

### Patients’ characteristics

A clinical distinction was made among niGAS isolates: niGAS isolates obtained from outpatients were classified as o-niGAS, whereas niGAS from hospitalized patients and/or emergency department admissions were classified as h-niGAS. Patients’ data about age, sex, comorbidities, viral co-infection, and trauma were collected at hospital admission. Comorbidities were classified according to the International Classification of Diseases (ICD-10-CM official guidelines for coding and reporting FY 2024 - UPDATED october 1, 2023 (October 1, 2023 - September 30, 2024), 2023). Trauma was defined as sudden tissue injury due to polytrauma, surgery, or blunt trauma. Concurrent viral infection was considered a risk factor. Corresponding outcomes and GAS complications occurring during hospitalization included length of stay, surgical debridement, bacterial superinfection, number of physician visits, 30-day mortality, intensive care unit (ICU) admission, and inflammatory biomarkers including neutrophils, leukocytes, and C-reactive protein (CRP) levels.

### Antibiotic susceptibility testing

Susceptibility to erythromycin, penicillin, and clindamycin was determined using the disk diffusion method according to Clinical and Laboratory Standards Institute (CLSI) guidelines (Clinical Laboratory Standards Institute, 2023). The reference strain *Streptococcus pneumoniae* ATCC49619 was used as a control, and inhibition zone diameters were measured and interpreted according to CLSI criteria (Clinical Laboratory Standards Institute, 2023).

### *emm* typing

*emm* gene typing was performed according to the Centers for Disease Control and Prevention (CDC) protocol (Frost et al., 2020). Briefly, DNA was extracted using the Qiagen DNA extraction kit (Qiagen, Germany), amplified through PCR with primers suggested by CDC, and, following column purification, sequenced with the M1a primer using BigDye^®^ Sequencing Kit. The sequence obtained was compared with the CDC database and assigned an *emm* type.

### Whole-genome sequencing and bioinformatic analysis

Library preparation and sequencing was done using MGI’s DNBSEQ™ technology: MGIEasyFS DNA Library Preparation Kit and MGIEasy Circularization Module were used. Sequencing was done with DNBSEQ-G400RS High-throughput Sequencing Kit (FCL PE150. 310 Cycles/Kit) in a DNBSEQ-G400 sequencing platform. Genomes were assembled using Spades (Bankevich et al., 2012) in the Galaxy-Australia platform (Galaxy Australia (Australian Biocommons and partners),), and the assembled genomes were annotated using Prokka (Seemann, 2014). Multi-locus sequence typing (MLST) was performed using AMRfinder plus in the Galaxy-Australia platform (Galaxy Australia (Australian Biocommons and partners), 2023). To determine the presence of the 27 SNPs that define the M1_UK_ lineage, genomes were analyzed using Snippy v4.6.0 (Seemann, 2015) and the reference MGAS5005 genome (GenBank accession: CP000017.2), screening the 27 variations previously reported by Lynskey and coworkers (Lynskey et al., 2019). To assess genetic differences among *emm1* isolates, whole-genome sequencing (WGS) data from isolates were compared with publicly available sequences from Latin American and global databases previously reported as lineages of M1_global_, M1_UK_, M1_DK_, and M1-ST1319 recently described in Argentina (Cipolla et al., 2025). The core SNP genome of the *emm1* isolates was aligned using the snippy-core module from Snippy together with the reference MGAS5005 genome. The recombinant regions were removed using Gubbins v3.3.5 (Croucher et al., 2015), and a phylogeny was constructed with GTR + CAT model in FastTree v2.1.11 (Price et al., 2010). Tree visualization, mid-point rooting, and annotation were done in iTOL v7.1.1 (Letunic and Bork, 2024). to detect outbreak-related genomic patterns. The phylogenetic tree including only the 13 outbreak isolates was performed using Realphy (Bertels et al., 2014). All genome sequences were deposited in the NCBI database under Bioproject No. PRJNA1260805.

### Statistical analysis

Categorical variables were compared using chi-square or Fisher’s exact test as appropriate. Continuous variables were assessed for normality and compared using Mann–Whitney or Wilcoxon test. A *p*-value <0.05 was considered statistically significant. Data analysis and visualization, including bar graphs, line graphs, and stacked area plots, were performed using Python (version 3.12.4), with the Pandas, NumPy, and Matplotlib libraries. Simpson Reciprocal Index (SRI) was calculated as the reciprocal of Simpson’s Index using Omnicalculator (https://www.omnicalculator.com/statistics/simpsons-diversity-index).

## Results

### Distribution of iGAS, niGAS, and clindamycin resistance

The study encompassed 2,307 isolates obtained between 2018 and 2024, of which 2,249 were niGAS and 58 were iGAS. Among niGAS, 2,119 isolates were o-niGAS (obtained from outpatients), and 130 isolates were h-niGAS (obtained from inpatients). Most h-niGAS were obtained from peritonsillar abscesses that required surgical intervention.

**Figure 1:**
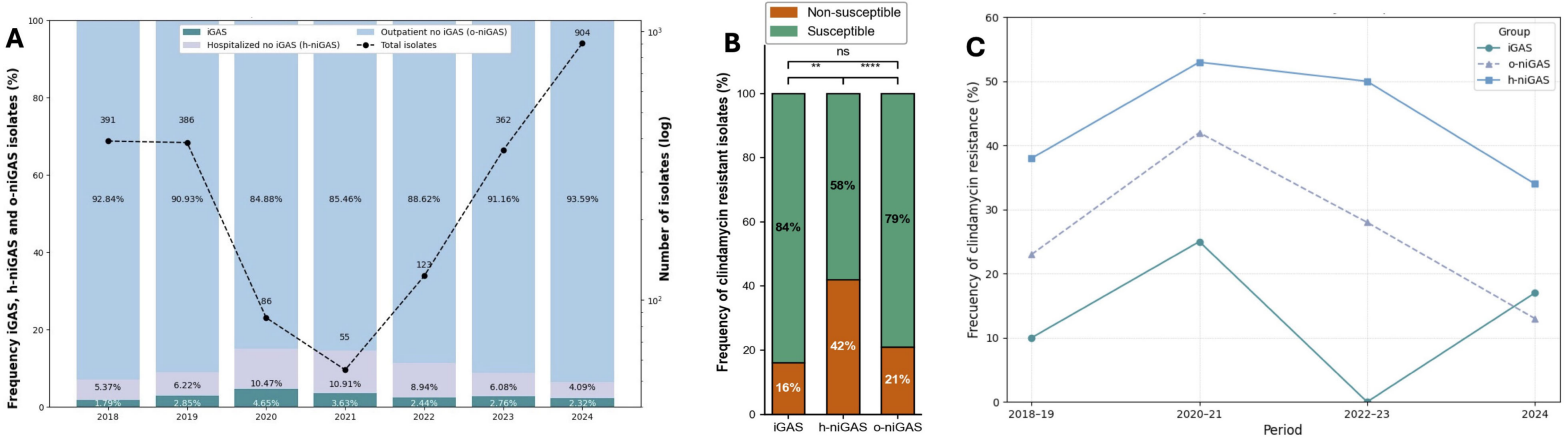
**(A)** Distribution of iGAS and niGAS during the period analyzed (2018–2024). Percentage of iGAS (blue), h-niGAS (green), and o-niGAS (light blue) isolates obtained each year. The dotted line graph with black circles represents the absolute number of GAS isolates obtained each year. **(B)** Distribution of clindamycin resistance by class of isolates. Percentage of clindamycin susceptible (light blue) and non-susceptible (green) isolates among iGAS, h-niGAS, and o-niGAS isolates. Non-susceptible includes resistant and intermediate, with intermediate isolates representing less than 1% of non-susceptible isolates. **(C)** Trend of clindamycin resistance by year for iGAS, h-niGAS, and o-niGAS. The significance of the difference between percentages was analyzed through Fisher’s exact test: ns, not significant; **p-value <0.005; ****p-value <0.00005.

The number of total GAS isolates obtained in the period 2018–2023 was approximately 400 per year, except for the period 2020–2021 when fewer than 100 cases were reported each year. In contrast, a substantial increase in the number of total GAS infections was observed in 2024, with 904 cases documented (**Figure 1A**). The h-niGAS isolates exhibited the highest percentage of clindamycin resistance, reaching 42%, whereas iGAS had the lowest clindamycin resistance at 16%. In fact, the clindamycin resistance of h-niGAS isolates was significantly higher than those of o-niGAS and iGAS isolates (**Figure 1B**). The o-niGAS isolates showed an intermediate rate of clindamycin resistance (21%). The clindamycin resistance rates of iGAS, h-niGAS, and o-niGAS remained consistent throughout the studied period, except for 2024, when iGAS exhibited increased clindamycin resistance (**Figure 1C**). More than 99% of clindamycin-resistant GAS isolates were also resistant to erythromycin, and all of the isolates analyzed were susceptible to penicillin (data not shown).

### *emm* type distribution among iGAS and h-niGAS

**Figure 2:**
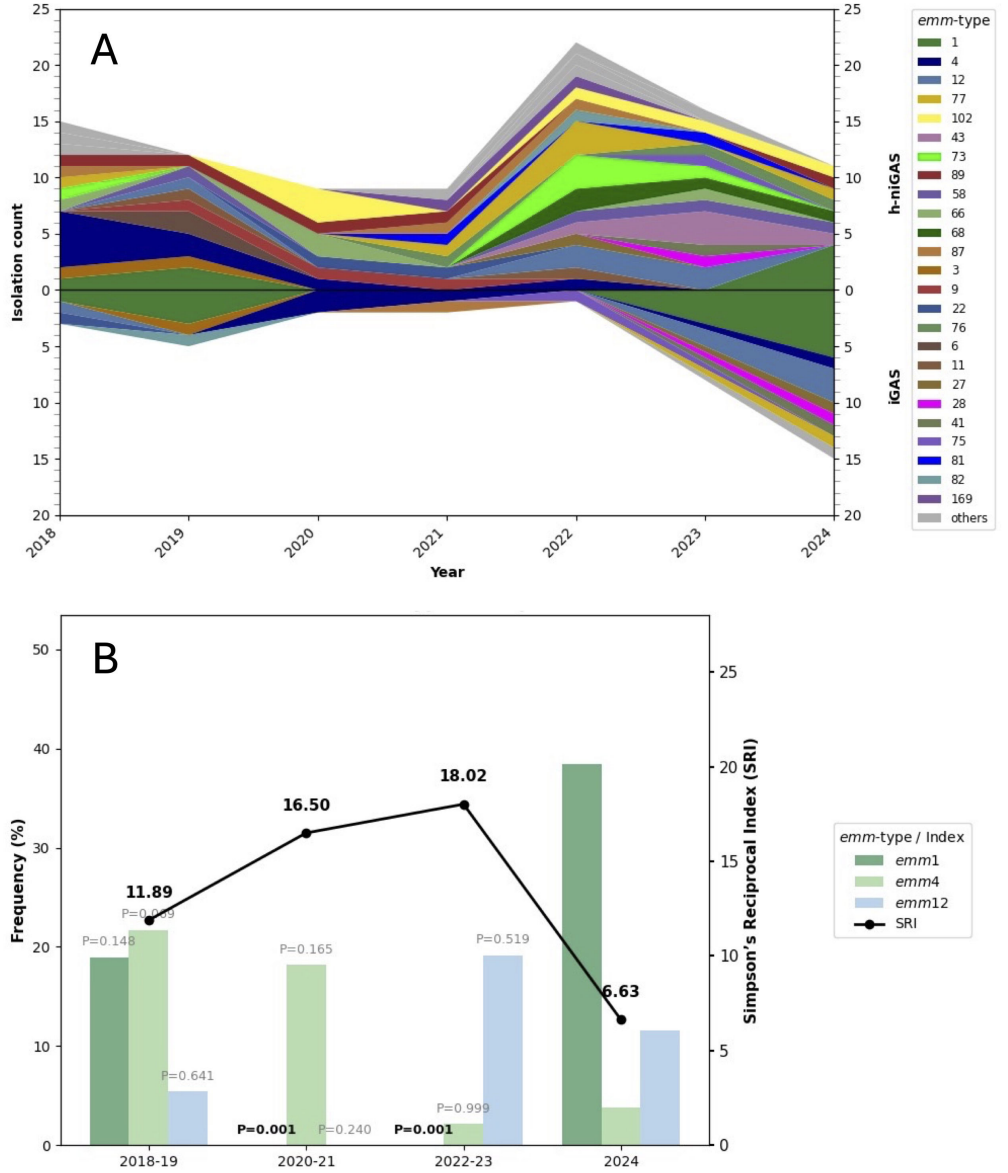
**(A)** *emm* type distribution in the period 2018–2024. All isolates included (*n* = 122) were iGAS and h-niGAS, the latter defined as non-invasive infections of hospitalized patients due to peritonsillar abscesses or other complications. Each *emm* type is represented with a particular color. “Other emm types” include *emm* types represented by one isolate (*emm74*, *emm164*, *emm48*, *emm480*, *emm49*, *emm94*, *emm131*, *emm161*, and *emm63*). **(B)** Simpson`s Reciprocal Index (SRI) and frequency of *emm1*, *emm12*, and *emm4.* SRI was calculated for each period (2018–2019, 2020–2021, 2022–2023, 2024). The frequency of the three most abundant *emm* types was calculated for the periods 2018–2019, 2020–2021, and 2022–2023 and compared to its frequency in 2024 using Fisher’s exact test. Significant differences in frequencies are shown in bold letters.

*emm* gene typing was performed in 48% (28/58) of iGAS and 72% (94/130) of h-niGAS; o-niGAS cases were not subjected to *emm* typing. Isolates that grew in culture after thawing were subjected to *emm* typing: 37/63 (59%) isolates in the period 2018–2019, 20/21 (95%) isolates in the period 2020–2021, 39/46 (85%) isolates in the period 2022–2023, and 26/58 (45%) isolates in 2024. Growth failure after thawing occurred randomly across all of the study periods and was not related with any specific clinical or epidemiological characteristic. Therefore, it does not introduce bias in the results of the study. The analysis identified 34 distinct *emm* types among the 122 isolates (**Figure 2A**). The most frequent *emm* type was *emm1*, comprising 13% of the isolates, followed by *emm4* and *emm12* with 10% and 7% of the isolates, respectively. Nine *emm* types were represented by a single isolate: *emm48*, *emm49*, *emm63*, *emm74*, *emm94*, *emm131*, *emm161*, *emm164*, and *emm480*. The frequency of types *emm1*, *emm4*, and *emm12* was calculated for the periods 2018–2019, 2020–2021, 2022–2023, and compared to 2024. A comparative analysis of frequency between periods showed a significant increase of type *emm1*, rising from 0% in 2020–2023 to 10% in 2024 (*p* < 0.001) (**Figure 2B**). A total of 18 new *emm* types, not detected in previous reports of our group (Wozniak et al., 2012; Wozniak et al., 2017), were found, and most of them surged after 2020. To estimate changes in diversity over time, the Simpson Reciprocal Index (SRI) was calculated to determine *emm* type diversity for the aforementioned periods. The SRI exhibited an upward trend until 2023, followed by a pronounced decrease in 2024, likely attributable to the expansion of type *emm1* (**Figure 2B**).

### Analysis of inpatient characteristics and clinical outcomes

A comparative analysis of demographic and epidemiologic characteristics, inflammatory markers, and clinical outcomes of inpatients with iGAS (*n* = 28) and h-niGAS (*n* = 94) infections was conducted (**Table 1**, left panel). The subsequent analysis revealed that type *emm1* was more frequently associated with iGAS cases (35.7% versus 7.5%, *p* < 0.01), whereas *emm4* and *emm12* were not. No statistically significant differences were observed between patients with iGAS and h-niGAS regarding age or gender. Patients diagnosed with iGAS exhibited a higher prevalence of at least one risk factor, including comorbidities, viral coinfection, and/or trauma (96.4% versus 26.2%, *p* < 0.01). Patients with iGAS infections exhibited a higher prevalence of underlying conditions, including chronic liver disease (35.7% versus 1.1%, *p* < 0.01; such as alcohol-induced cirrhosis, non-alcoholic steatohepatitis, liver transplantation, and hepatic metastases) and immunosuppression (21.4% versus 6.5%, *p* = 0.019; namely, cancer, juvenile idiopathic arthritis/rheumatoid arthritis, systemic lupus erythematosus, and HIV infection). Furthermore, blunt trauma and polytrauma were identified as triggering factors in 21.4% of iGAS cases compared to 1.1% in h-niGAS cases (*p* < 0.01).

**Table 1:** *emm* type, patients’ characteristics, and clinical outcomes of iGAS and h-niGAS obtained in 2024 compared to isolates obtained in previous years.

	Total isolates of the period 2018–2024 (n = 122)			iGAS					h-niGAS			
	iGAS (n = 28)	h-niGAS (n = 94)	P-value	2024 (n = 15)		2018–23 (n = 13)		P-value	2024 (n = 11)		2018–23 (n = 83)	P-value
GAS *emm* type												
*emm*1, *n* (%)	**10 (35.7%)**	**7 (7.5%)**	**<0.001**	6 (40%)	4 (30.8%)		0.7055		**4 (36.4%)**	**3 (3.61%)**		**0.0031**
*emm*4, *n* (%)	4 (14.2%)	5 (5.3%)	0.11	1 (6.6%)	3 (23.1%)		0.3111		0 (0%)	9 (10.84%)		0.5921
*emm*12, *n* (%)	4 (14.2%)	9 (9.6%)	0.48	3 (20%)	1 (27%)		0.6000		0 (0%)	5 (6.02%)		>0.999
Patients’ characteristics												
Age, median (range)	29 (1–89)	23 (1–90)	0.0693	49 (4–89)	18 (1–82)		0.0716		34 (5–60)	23 (1–90)		0.3508
Male, *n* (%)	18 (64.3%)	50 (53.2%)	0.387	9 (60%)	9 (69.2%)		0.7055		6 (54.5%)	44 (53.01%)		>0.999
Risk factors												
Comorbidities^a^, *n* (%)	16 (57.1%)	17 (18.1%)	>0.9999	12 (80%)	10 (76.9%)		>0.9999		1 (9.1%)	17 (20.48%)		0.6843
Chronic liver disease	**10 (35.7%)**	**1 (1.1)**	**<0.001**									
Immunosupression	**6 (21.4%)**	**6 (6.5%)**	**0.019**									
Viral coinfection	**10 (35.7%)**	**2 (2.3%)**	**<0.001**	5 (33.3%)	5 (38.5%)		>0.9999		0 (0%)	2 (2.41%)		>0.999
Trauma	**6 (21.4%)**	**1 (1.1)**	**<0.001**	4 (26.7%)	2 (15.4)		0.6546		0 (0%)	0 (0%)		>0.999
≥1 risk factor	**27 (96.4%)**	**19 (20.21%)**	**<0.001**	14 (93.3%)	13 (100%)		>0.9999		1 (9.09%)	19 (22.9%)		0.4470
Inflammatory markers												
Neutrophils, median (range)	**10,895** **(7,195–19,267)**	**8,390** **(6,350–11,590)**	**0.039**	11,420 (2,160–27,000)	3,270 (3,210–26,660)		0.9402		10,890 (1,700-18,310)	8,130 (1,790–25,330)		0.1321
White blood cells, median (range)	12,750 (8,240–21,450)	11,150 (9,000–14,500)	0.29	13,600 (1,400–31,000)	12,000 (4,400–29,000)		0.4064		14,000 (1,400-22,300)	11,000 (4,500–29,000)		0.4738
C-reactive protein, median (range)	**18.5** **(1–43)**	**6.75** **(0.58–38.6)**	**<0.001**	16 (1–42)	24 (3.4–43)		0.4805		10 (3-22)	6.2 (0.58–38.6)		0.2782
Clinical outcomes												
Stay (days), median (range)	**8.5 (5.5–20)**	**2 (1–3)**	**<0.001**	8 (3–25)	9 (2–54)		0.4447		3 (2-9)	2 (1–21)		0.5870
Surgery, *n* (%)	10 (35.7%)	36 (38.3%)	0.8	11 (73.3%)	7 (53.8%)		0.4328		**11 (100%)**	**47 (56.63%)**		**0.0058**
Bacterial superinfection, *n* (%)	7 (25.0%)	12 (12.8%)	0.117	5 (33.3%)	2 (15.4%)		0.3955		3 (27.3%)	9 (10.84%)		0.1452
Number of visits, mean (range)	1 (1–2)	1 (1–2)	0.45	1 (1–2)	2 (1–3)		0.0512		**1.73 (1-3)**	**1.29 (1–3)**		**0.0254**
30-day mortality, *n* (%)	**2 (7.1%)**	**0 (0%)**	**<0.001**	1 (6.7%)	7 (7.7%)		>0.9999		0 (0%)	0 (0%)		>0.999
ICU admission, *n* (%)	**15 (53.6%)**	**7 (7.4%)**	**<0.001**	7 (46.7%)	8 (61.5%)		0.4757		0 (0%)	7 (8.43%)		>0.999

*P*-values were calculated using Fisher’s exact test (95% confidence interval). For comparisons with *p* < 0.05, values and cells are indicated in bold.

a Including immunosupression, cancer, chronic liver disease, hypertension, asthma, and obesity.

Concurrent viral infection was significantly more frequent among iGAS than h-niGAS cases (35.7% versus 2.3%, *p* < 0.01). The triggering viruses included cases of influenza (*n* = 3), SARS-CoV-2 (*n* = 2), rhinovirus/enterovirus (*n* = 2), human metapneumovirus (*n* = 2), parainfluenza (*n* = 1), varicella-zoster virus (*n* = 1), and molluscum contagiosum (*n* = 1). Additionally, patients with iGAS exhibited significantly higher levels of CRP (*p* < 0.01) and higher neutrophil counts (*p* = 0.039) at diagnosis. The clinical outcomes for patients with iGAS were more severe than those with h-niGAS, which was reflected in a more extended hospital stay, higher rates of intensive care admission, and increased 30-day mortality.

The iGAS cases of the 2024 outbreak exhibited no distinguishable characteristics in terms of *emm* type distribution, age, gender, associated risk factors, clinical outcomes, and inflammatory markers when compared to iGAS from previous years (**Table 1**, central panel). In contrast, the frequency of *emm1* isolates was significantly higher in h-niGAS isolates obtained in 2024 compared to those obtained in the 2018–2023 period (36.4% versus 3.6%, *p* = 0.0031) (**Table 1**, right panel). Furthermore, h-niGAS cases observed in 2024 exhibited a significantly higher prevalence of severe clinical outcomes as indicated by the number of surgeries required (100% versus 56.6%, *p* = 0.005) and the number of visits (1.7 versus 1.3, *p* = 0.025). These findings suggest that iGAS occurring during 2024 were not different from iGAS occurring in previous years. In contrast, h-niGAS in 2024 were more frequently caused by *emm1* than in previous years and had more severe clinical outcomes.

### Genomic analysis of inpatients’ invasive isolates obtained during the 2024 outbreak

**Figure 3:**
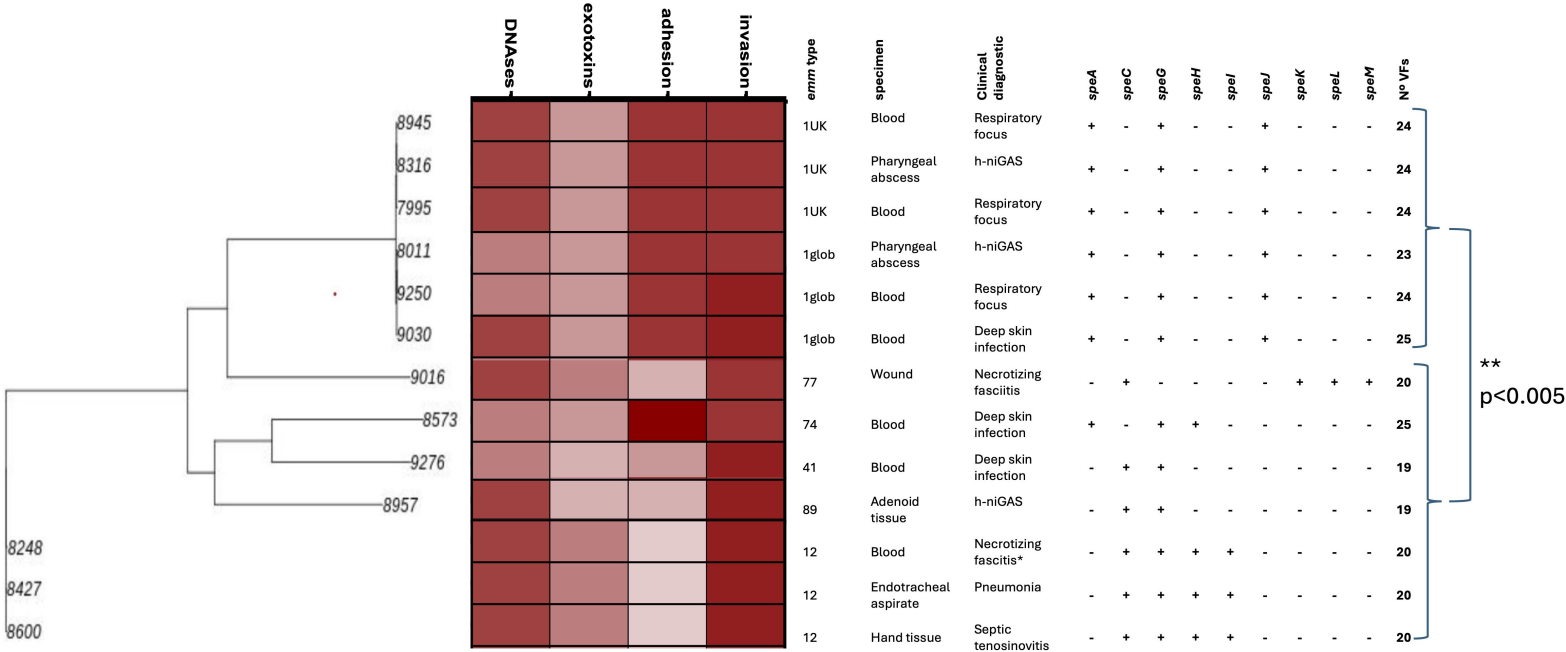
Clinical and genetic characteristics of iGAS and h-niGAS outbreak isolates analyzed through WGS. A phylogenetic tree was constructed from core SNPs using Realphy software (24). The VF genes coding for DNAses were *mf/spd*, *mf2*, *mf3*, and *sda*. The VF genes coding for exotoxins were *ssa*, *smeZ*, *speA*, *speB*, *speC*, *speG*, *speH*, *speI*, *speJ*, *speK*, *speL*, and *speM*. The VF genes associated with adhesion were *fbaA*, *fbaB*, *fbp54*, *fctA*, *fctB*, *grab*, *cpa*, *lmb*, *sipA*, and *srtC1*. The VF genes associated with invasion were *hasABC*, *hylP*, *ska*, *scpA*, *sic*, *ides/Mac*, and *slo*. All isolates had the superantigen genes *speB* and *smeZ*. The color intensity is proportional to the percentage of VFs that each isolate has. The number of VFs was calculated as the sum of VFs in the four categories. The number of VFs associated with *emm1* isolates and non-*emm1* were compared using Student’s *t*-test.

To further analyze the outbreak isolates, 10 iGAS and three h-niGAS isolates obtained in 2024 were subjected to whole-genome sequencing. Among iGAS, four were *emm1*, three were *emm12*, and three were *emm74*, *emm77*, and *emm41*, respectively. Among h-niGAS, two were *emm1* and one was *emm89.* All of the *emm1* isolates were ST28, the most frequently associated ST, and all of the *emm12* isolates were ST36, a frequent ST among *emm12* isolates (Enright et al., 2001). A genetic analysis of *emm1* iGAS isolates revealed that two of them belonged to the M1_UK_ lineage. This classification was confirmed by the presence of 27 SNPs, which is a characteristic of the M1_UK_ lineage. The phylogenetic tree based on core SNPs of the 13 outbreak isolates grouped the *emm1* isolates in one clade and the *emm12* isolates in a different clade (**Figure 3**). Virulence factors (VFs) were screened in all isolates according to the Virulence Factor Database (VFDB) (Chen et al., 2005). Notably, *emm1* isolates had a significantly higher number of genes coding for VFs compared to non-*emm1* isolates (**Figure 3**). The VFs were grouped into four categories: DNAses, exotoxins, and adhesion-associated and invasion-associated factors. The VF genes coding for DNAses were *mf/spd*, *mf2*, *mf3*, and *sda*. The VF genes coding for exotoxins were *smeZ*, *speA*, *speB*, *speC*, *speG*, *speH*, *speI*, *speJ*, *speK*, *speL*, and sp*eM.* The VF genes involved in adhesion were *fbaA*, *fbaB*, *fbp54*, *fctA*, *fctB*, *grab*, *cpa*, *lmb*, *sipA*, and *srtC1*. The VF genes involved in invasion were *hasABC*, *hylP*, *ska*, *scpA*, *sic*, *ides/Mac*, and *slo*. The most notable distinction between the VFs of *emm1* and non-*emm1* isolates pertained to the genes involved in adhesion: *emm1* isolates had the entire array of 10 genes, whereas the other *emm* types had only two to five adhesion-associated genes. Genes exclusively present in *emm1* isolates were *grab*, a protein-G-related alpha2M-binding protein that controls proteolysis in the surface of GAS and has a role in adhesion (Rasmussen et al., 1999), and *fctA*, *fctB*, *sipA*, and *srtC*, coding for major pilin, minor pilin, pilin signal peptidase, and pilin sortase, respectively, that are involved in pili formation, a major adhesin of GAS (Tsai et al., 2024). It is worth mentioning that one h-niGAS was M1_UK_ and had also the entire array of genes coding for adhesion-related proteins. Genes coding for *speA* and *speJ*, two superantigen toxins associated with iGAS (Alcolea-Medina et al., 2023), were exclusively present in *emm1* isolates. SpeA was also present in the *emm74* isolate. In contrast, *speC*, a superantigen toxin present in the M1_DK_ lineage (Johannesen et al., 2023) and associated with virulence, was present in *emm12*, *emm89*, *emm41*, and *emm77* isolates. Genes involved in virulence factor regulation were analyzed: *covR/S* (influences the transcription of most virulence genes), *rocA* (regulator of *covR/S*), and *ropB* (associated with invasiveness) (Ferretti et al., 2022). Only one iGAS isolate (#9016 in **Figure 3**) had a putative mutation in *covR* (M170I), whereas genes *covS*, *rocA*, and *ropB* had a wild-type sequence. These results show that virulence is more associated with *emm* type rather than with the invasive or non-invasive nature of the isolates.

### Phylogenetic analysis of *emm1* outbreak in inpatients’ isolates

**Figure 4:**
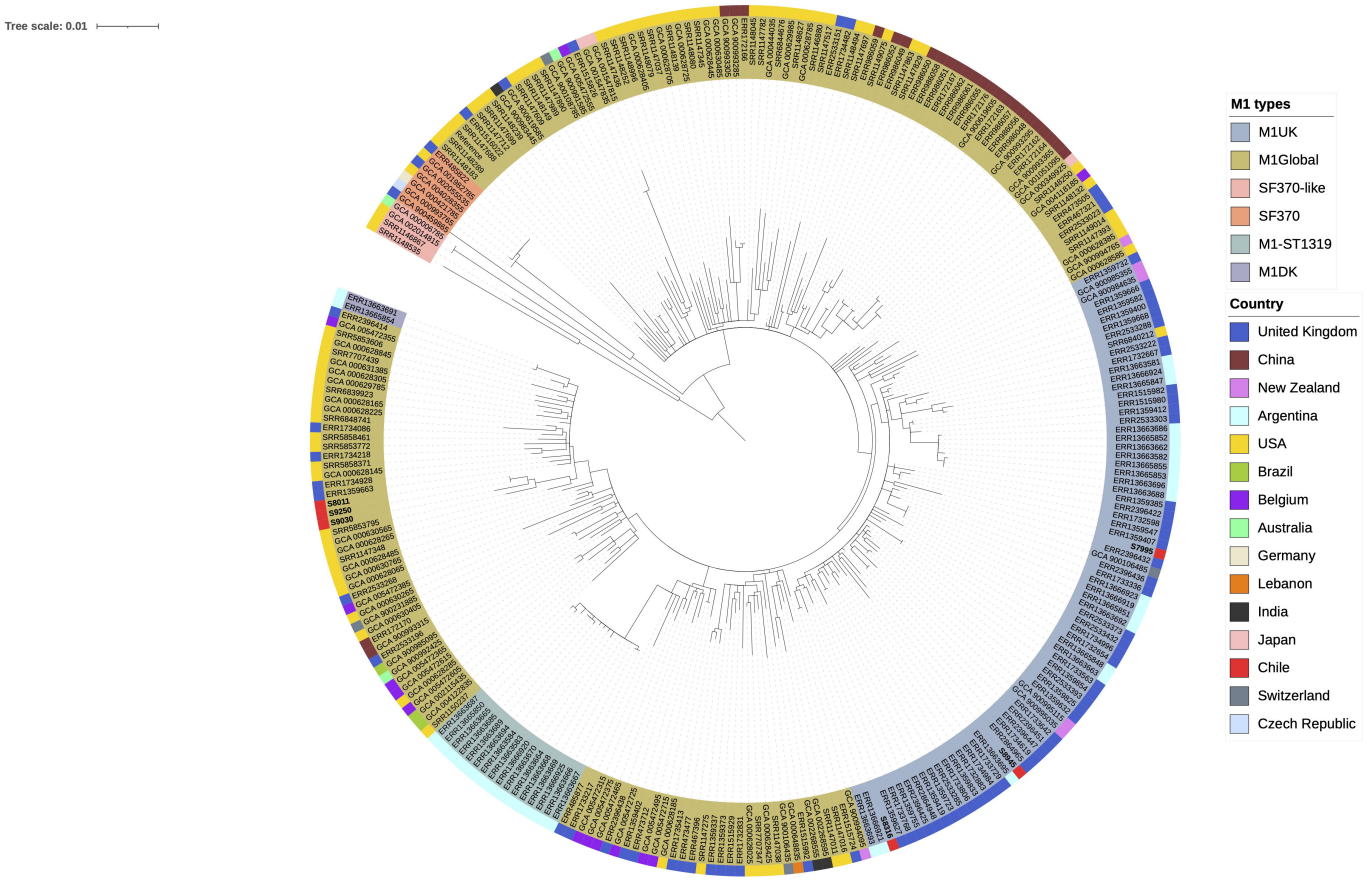
Phylogenetic tree constructed from core single-nucleotide polymorphisms of *emm1* isolates obtained from 15 different countries. SF370 lineage is one of the first fully sequenced *emm1* genome, and M1-ST1319 is an Argentinian clade of M1_global_ genome that belongs to ST1319, in contrast to most M1_global_ isolates that belong to ST28. The *emm1* genomes included in the tree were from global and Latin American public databases.

A phylogenetic analysis of *emm1* isolates showed that M1_UK_ and M_global_ formed defined clades (**Figure 4**). The SF370 lineage is one of the first fully sequenced strains of M1 and provides the context of modern M1 types. M1_global_ strains formed two clades; one of them contained the Argentinian clone M1-ST1319, and the other clade contained the SF370 lineage in a defined sub-clade. Chilean M1_UK_ isolates do not form a separate clade and were close to M1_UK_ isolates from United Kingdom and Argentina (**Figure 4**). In contrast, Chilean M1_global_ isolates are clustered together but not defining a specific clade and are close to isolates from United States and United Kingdom. These results show that no changes occurred in the Chilean isolates with respect to the isolates from United Kingdom or United States and also give insights that the introduction of these strains could have occurred from these countries.

## Discussion

A substantial upsurge in iGAS was documented during the first 6 months of 2024 in Chile. This outbreak most likely resulted from an increase in the total number of cases, not specifically iGAS cases.

The high clindamycin resistance levels in h-niGAS pose a significant challenge because these infections frequently require treatment with clindamycin. The increased consumption of antibiotics, specifically macrolides, prescribed for treating secondary infections in patients with SARS-CoV-2 during 2020–2021, may explain the increased clindamycin resistance levels (Allel et al., 2023; Patel et al., 2023). On the other hand, the low clindamycin resistance in iGAS offers a potential advantage in the treatment of severe infections.

It is well documented that *emm* type distribution varies with socioeconomic conditions and geographical location: in low-income countries, there are no dominant *emm* types and a higher diversity is observed. Moreover, many of the common *emm* types in high- and medium-income countries are less common in low-income countries (Steer et al., 2009). This differential *emm* type distribution can even be observed within a single country: a higher *emm* type diversity was reported among GAS isolates of patients from informal settlements with respect to GAS from high-income patients in Brazil (Tartof et al., 2010). In the present study, *emm* type distribution is highly polyclonal compared to the oligoclonal distribution reported in previous studies: the diversity reported in 2012 and 2017 had a calculated SRI value of 11.04 and 13.4, respectively (Wozniak et al., 2012; Wozniak et al., 2017), whereas the average SRI for the period 2018–2023 was 15.47. Moreover, 18 novel *emm* types, not reported in the previous works, were detected. These findings suggest that the 2024 outbreak was preceded by an increase in *emm* type diversity. One potential factor contributing to this observed change in Chile is the significant increase in immigration, which rose from less than 500,000 individuals before 2015 to over 1,900,000 in 2023. This influx of migrants, primarily from countries such as Colombia and Venezuela, as reported by the National Service of Migration of Chile (National Service of Migration, [[NoYear]]), may have played a role in shaping the observed changes in *emm* type distribution. The variation in *emm* type diversity could decrease the potential coverage of multivalent M-protein-based vaccines—for example, Mx10 vaccine is based on the 10 most frequent *emm* types in Chile (Wozniak et al., 2018) and has a coverage of 82% of circulating *emm* types according to previous reports (Wozniak et al., 2012; Wozniak et al., 2017). However, coverage would be only 48% if the current *emm* type distribution is considered. For this reason, vaccines based in conserved antigens appear to be more appropriate for this scenario. In fact, this type of candidate vaccine has shown good immunogenicity and protective efficacy in animal models (Bi et al., 2019; Rivera-Hernandez TRhyme et al., 2020).

According to the results of this work, iGAS were significantly associated with several riskfactors such as comorbidities, trauma, or concomitant viral infection, had more severe clinicaloutcomes and inflammatory markers, and had increased frequency of type *emm1.* The association of comorbidities with iGAS, particularly immunosuppression, is an expected issue. Indeed a study revealed a significant association between immunosuppression and ICU mortality, with a fourfold increase observed in patients with iGAS infections (Orieux et al., 2024). However, the role of hepatic disease as a risk factor remains to be thoroughly investigated. According to some reports, the presence of liver dysfunction or obesity has been demonstrated to pose a significant risk of severity of iGAS infection (Mehta et al., 2006; Langley et al., 2016). Influenza co-infection was also expected according to findings reported in the literature (Lassoued et al.,; de Gier et al., 2019). Notably, viruses other than influenza were associated as triggering factors of iGAS. Collectively, these findings show that *emm1* GAS, in conjunction with comorbidities, viruses, and trauma, may precipitate more severe manifestations of the disease. We observed that iGAS from the outbreak were not different from previous iGAS, whereas h-niGAS from the outbreak were different from previous ones in terms of higher frequency of type *emm1* and severity of clinical outcomes, underscoring the importance of h-niGAS in the outbreak. The higher frequency of type *emm1* during the outbreak is in accordance with the higher number of VFs, suggesting an explanation for its high frequency in human infections. The decrease of *emm4*, an *emm* type frequently associated to niGAS (Rampersadh et al., 2024), goes in the same direction. The fact that only one of the iGAS isolates obtained in 2024 has a mutation in the CovRS system, the main factor associated with increased virulence in iGAS, reinforces the notion that the outbreak is associated to an expansion of type *emm1* rather than an increase in virulence of the circulating strains.

The main limitation of our study is the small sample size due to the participation of only one laboratory of microbiology in Chile. Moreover, not all hospital-derived strains could be studied, as several did not grow upon subculture. The strengths of our study lie in the comprehensive documentation of demographic and clinical characteristics of GAS-infected patients that were integrated with the microbiological and molecular characteristics of iGAS and h-niGAS infections, resulting in a multi-point-of-view characterization of the outbreak. This study provides valuable insights into the genomic epidemiology of iGAS in Chile and highlights the importance of conducting genomic surveillance to guide future research that could contribute to iGAS control strategies.

## Data Availability

The datasets presented in this study can be found in online repositories. The names of the repositories and accession numbers can be found in the article by contacting the authors.
